# Resveratrol Ameliorated Hyperlipidemia: Association with Modulation of the Gut Microbiota and Bile Acid Metabolism

**DOI:** 10.3390/nu18142384

**Published:** 2026-07-21

**Authors:** Ruiying Luo, Qing Peng, Su Wu, Shuwen Liu, Chan Wang

**Affiliations:** 1 NMPA Key Laboratory for Research and Evaluation of Drug Metabolism, Guangdong Provincial Key Laboratory of New Drug Screening, School of Pharmaceutical Sciences, Southern Medical University, Guangzhou 510515, China; 14749776792@163.com (R.L.); 13762043750@163.com (S.W.); liusw@smu.edu.cn (S.L.); 2 School of Pharmaceutical Sciences, South-Central Minzu University, Wuhan 430074, China; pq714929@163.com

**Keywords:** resveratrol, hyperlipidemia, gut microbiota, bile acids

## Abstract

**Background:** Hyperlipidemia represents a pivotal risk factor for multiple cardiovascular and metabolic diseases, creating an urgent demand for safe, effective novel therapeutic interventions. Resveratrol (RES), a natural plant polyphenol, has exhibited great potential as a hypolipidemic agent for hyperlipidemia treatment. **Methods:** In this work, we established a high-fat diet (HFD)-induced hyperlipidemic mouse model to systematically investigate the lipid-regulating efficacy of RES. **Results:** Oral administration of RES significantly decreased serum levels of total cholesterol and low-density lipoprotein cholesterol, while markedly increasing high-density lipoprotein cholesterol concentrations. Histopathological analyses further demonstrated that RES alleviated HFD-induced hepatic and ileal injury. Moreover, RES significantly decreased the circulating levels of several bile acids (BAs), including taurodeoxycholic acid, taurolithocholic acid, lithocholic acid, and tauro-(α + β)-muricholic acid. RES also ameliorated HFD-induced gut microbial dysbiosis, as evidenced by a reduction in the *Firmicutes/Bacteroidetes* ratio, increased relative abundances of *non_f_Muribaculaceae*, *Lactobacillus*, *Ileibacterium*, and *Bacteroides*, and decreased abundances of *Lachnospiraceae_NK4A136_group* and *unclassified f_Lachnospiraceae*. Spearman correlation analysis revealed significant associations between gut microbial taxa and BA profiles, suggesting that coordinated regulation of the gut microbiota and BA metabolism contributes to the hypolipidemic effects of RES. **Conclusions:** Collectively, RES alleviates HFD-induced hyperlipidemia by remodeling gut microbiota composition and restoring bile acid metabolic homeostasis.

## 1. Introduction

With rapid socioeconomic development, profound changes in lifestyle and dietary habits have adversely affected human health, making hyperlipidemia one of the most prevalent chronic metabolic disorders worldwide [1]. Hyperlipidemia is characterized by elevated serum concentrations of total cholesterol (TC), triglycerides (TG), and low-density lipoprotein cholesterol (LDL-C) [1], and is closely associated with the development of numerous diseases, particularly hepatic and cardiovascular disorders [2,3]. Current clinical strategies for the management of hyperlipidemia primarily include lifestyle interventions, pharmacological therapies, and nutritional supplementation. Lipid-lowering agents such as statins and fibrates are widely prescribed because of their rapid and potent effects on serum lipid profiles; however, long-term administration may be accompanied by adverse effects. Consequently, natural bioactive compounds derived from foods and medicinal plants have attracted increasing attention because of their favorable safety profiles and moderate lipid-regulating activities. These compounds may represent promising alternatives for the long-term management of hyperlipidemia. For example, dietary fiber supplementation has been shown to contribute to cholesterol homeostasis and improve lipid metabolism [4].

Bile acids (BAs) are endogenous metabolites of cholesterol and major constituents of bile that play essential roles in lipid digestion and absorption. The liver is the exclusive site of BA synthesis. Under physiological conditions, cholesterol is primarily converted into primary BAs through the classical synthetic pathway. These primary BAs are subsequently conjugated with glycine or taurine and further transformed into secondary BAs by gut microbial enzymes [5]. In addition to facilitating fat digestion and absorption, microbially modified BAs serve as important signaling molecules that influence gut microbial composition and function [6]. The reciprocal interaction between gut microbiota and BAs plays a central role in energy homeostasis, lipid metabolism, and cholesterol regulation [7]. Therefore, dysregulation of the gut microbiota–BA axis may contribute substantially to the development of hyperlipidemia and related metabolic disorders, making this axis an attractive therapeutic target.

Resveratrol (RES), a naturally occurring polyphenolic compound, exhibits a wide range of pharmacological properties, including antioxidant, anti-inflammatory, and immunomodulatory activities [8]. In particular, accumulating evidence supports its beneficial effects in the prevention and treatment of chronic metabolic disorders, including hyperlipidemia, hyperglycemia, and hypertension [9]. Recent studies have demonstrated that RES can modulate gut microbiota composition and alter microbial metabolites, including BAs, short-chain fatty acids, and luminal lipids, thereby alleviating manifestations of metabolic syndrome [10]. Based on these observations, we hypothesized that long-term dietary supplementation with RES may exert beneficial effects on hyperlipidemia through the regulation of gut microbiota and BA metabolism. Therefore, the present study aimed to investigate the therapeutic effects of RES on HFD-induced hyperlipidemia in mice, with particular emphasis on the interactions between gut microbiota and BA metabolism. Targeted metabolomics and 16S rRNA gene sequencing were employed to characterize BA profiles and gut microbial composition in hyperlipidemic mice. These findings may provide mechanistic insights into the lipid-lowering mechanisms of RES and establish a theoretical foundation for its application in the prevention and treatment of hyperlipidemia.

## 2. Materials and Methods

### 2.1. Materials

Resveratrol (RES) powder was purchased from Shanghai Aladdin Biochemical Technology Co., Ltd. (Shanghai, China). Simvastatin (batch No. H19980054) was obtained from Shanghai Xinyi Wanshang Pharmaceutical Co., Ltd. (Shanghai, China). Commercial serum lipid detection kits were supplied by Nanjing Jiancheng Bioengineering Institute (Nanjing, China). High-fat diet containing 60% fat was bought from Guangzhou Jinwei Biotechnology Co., Ltd. (Guangzhou, China). All other chemical reagents used in this study were of analytical grade.

### 2.2. Animal Experiments

Animals. Sixty male C57BL/6J mice weighing 18–22 g were obtained from the Guangdong Medical Experimental Animal Center (Guangzhou, Guangdong Province, China) and housed under specific pathogen-free conditions at the Experimental Animal Center of Southern Medical University. All animal procedures were approved by the Experimental Animal Ethics Committee of Southern Medical University (Approval No. SMUL202412009 and Approval Date: 15 January 2025).

**Figure 1:**
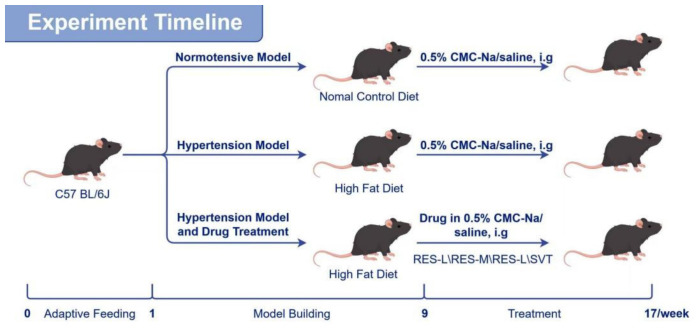
Schematic illustration of the animal experimental design (created using Figdraw 2.0).

HFD-induced hyperlipidemia. Following a 1-week acclimatization period, 10 mice were randomly assigned to the normal control (NC) group and fed a standard chow diet, whereas the remaining 50 mice were fed a HFD for nine consecutive weeks to establish a hyperlipidemia model. After successful model establishment, an eight-week treatment period was initiated. The HFD-fed mice were randomly allocated into five groups (*n* = 8): NC group: standard diet + 0.5% sodium carboxymethyl cellulose (CMC-Na) (10 mL/kg/day); HC group: HFD + 0.5% CMC-Na (10 mL/kg/day); RES-L group: HFD + low-dose RES (50 mg/kg/day); RES-M group: HFD + medium-dose RES (100 mg/kg/day); RES-H group: HFD + high-dose RES (200 mg/kg/day); and STV group: HFD + simvastatin (10 mg/kg/day) as the positive control group (Figure 1). Following the final administration, mice were fasted for 12 h with free access to water. On the following day, animals were anesthetized with sodium pentobarbital, and blood samples were collected from the abdominal aorta. Subsequently, liver and ileal tissues were harvested immediately and stored at −80 °C for subsequent analyses.

### 2.3. Biochemical Analysis of the Serum Samples

Serum biochemical analyses were performed to determine TC, TG, high-density lipoprotein cholesterol (HDL-C), and LDL-C levels. All measurements were conducted using commercial assay kits strictly according to the manufacturers’ instructions.

### 2.4. Histopathological Examination

To evaluate hepatic vacuolation, inflammatory infiltration, and lipid accumulation, tissue samples were collected from the right lobe of the liver and fixed in 4% paraformaldehyde. Liver sections were subsequently subjected to hematoxylin and eosin (H&E) staining to assess histological structure and inflammatory changes, and Oil Red O (ORO) staining to evaluate lipid deposition. Simultaneously, ileal tissues were fixed in 4% paraformaldehyde and stained with H&E for histopathological evaluation. Histological images were captured using a microscope (ECLIPSE Ti-S, Nikon Corporation, Tokyo, Japan) and analyzed for pathological structural changes.

### 2.5. Quantitative Real-Time Polymerase Chain Reaction (qRT-PCR) Analysis

Total RNA was extracted from liver and ileal tissues using an RNA isolation kit (Foregene, Chengdu, China) according to the manufacturer’s instructions. The isolated RNA was reverse-transcribed into complementary DNA using a PrimeScript RT reagent kit (Vazyme, Nanjing, China). Relative mRNA expression levels of target genes were quantified using the 2^−ΔΔCT^ method as previously described by Chen et al. (2025) [11]. The primer sequences used for qRT-PCR analysis are listed in Table 1.

### 2.6. Targeted Quantification of Serum BAs

Blood samples were centrifuged at 4000× *g* for 10 min to obtain serum samples. Briefly, 50 μL of serum was mixed with 150 μL of methanol, vortexed for 2 min, and centrifuged at 20,000× *g* for 10 min at 4 °C. The resulting supernatant was concentrated under vacuum to approximately 160 μL, and the residue was reconstituted with an acetonitrile-water solution to a final volume of 40 μL. Prepared samples were analyzed using an ultra-performance liquid chromatography–tandem mass spectrometry (UPLC–MS/MS) system (Waters Corp., Milford, MA, USA), with chromatographic and mass spectrometric conditions established according to a previously reported method [12]. Quantitative BA profiling was performed by Suzhou Panomike Biomedicine Technology Co., Ltd. (Suzhou, China).

### 2.7. Analysis of Gut Microbiota Composition

During the final week of the experiment, fresh fecal samples were obtained under aseptic conditions. All fecal specimens were subsequently stored in a −80 °C refrigerator for subsequent analysis. The fecal of mice were thawed, and microbial genomic DNA was extracted using the E.Z.N.A.^®^ Mag-Bind Stool DNA Kit (M4016, Omega Bio-tek, Norcross, GA, USA). DNA concentration and purity were evaluated by 1% agarose gel electrophoresis. The V3–V4 hypervariable regions of the bacterial 16S rRNA gene were amplified using primer pair 338F (5′-ACTCCTACGGGAGGCAGCAG-3′) and 806R (5′-GGACTACHVGGGTWTCTAAT-3′) [13]. Purified PCR products were used to construct sequencing libraries using the NEXTFLEX Rapid DNA-Seq Kit (Bioo Scientific, Austin, TX, USA). Sequencing was performed on the Illumina PE300/PE250 platform (Illumina, San Diego, CA, USA) by Majorbio Bio-Pharm Technology Co., Ltd. (Shanghai, China). Bioinformatic analyses of gut microbiota composition were conducted using the Majorbio Cloud Platform (https://cloud.majorbio.com, accessed on 1 July 2025).

### 2.8. Statistical Analysis

Data are presented as the mean ± standard error of the mean (SEM). Statistical analyses were performed using Statistical Package for Social Sciences version 23.0 (Chicago, IL, USA). Comparisons among multiple groups were conducted using one-way analysis of variance followed by Tukey’s post hoc test. Statistical significance was defined as * *p* < 0.05, ** *p* < 0.01, *** *p* < 0.001 and **** *p* < 0.0001. All figures were generated using GraphPad Prism version 9.3.0 (GraphPad Software, San Diego, CA, USA).

## 3. Results

### 3.1. Effects of RES on Body Weight and Serum Lipid Levels in Hyperlipidemic Mice

**Figure 2:**
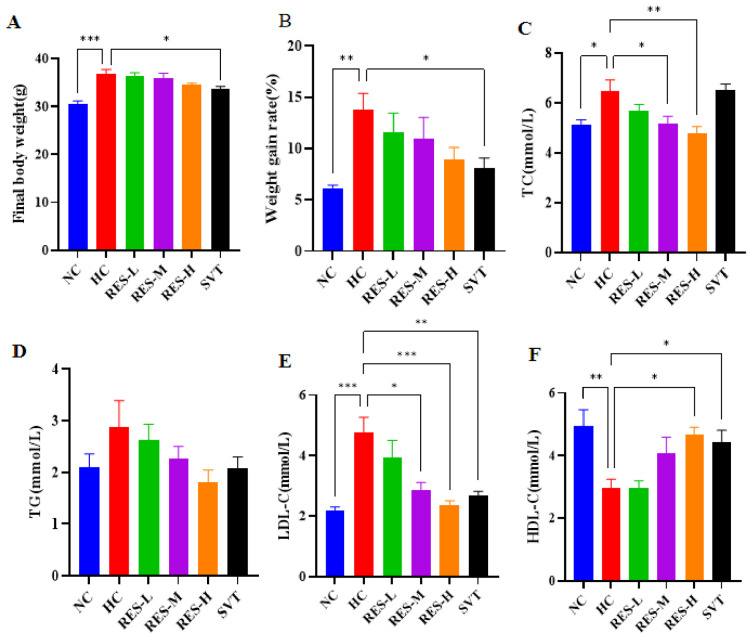
Effects of resveratrol on body weight and lipid metabolism in hyperlipidemic mice. (**A**) Final body weight of mice in each group. (**B**) Body weight gain following treatment administration. (**C**–**F**) Serum levels of total cholesterol, triglycerides, low-density lipoprotein cholesterol, and high-density lipoprotein cholesterol. Data are presented as mean ± standard error of the mean (*n* = 6). * *p* < 0.05, ** *p* < 0.01, and *** *p* < 0.001.

During the experimental period, mice were fed either a normal diet or a HFD for 17 weeks. At the end of the experiment, mice in the HC group exhibited significantly greater body weight gain than those in the NC group (Figure 2A). Compared with the HC group, all treatment groups showed varying degrees of attenuation in body weight gain, with the most pronounced reductions observed in the NC and SVT groups (Figure 2B). These findings indicated that RES exerted anti-obesity effects in HFD-fed mice, which is consistent with previous findings reported by Qiao et al. [14], who proposed that RES regulates body weight gain through modulation of the gut microbiota. Compared with the NC group, mice in the HC group exhibited significantly elevated serum TC and LDL-C levels (Figure 2C,E), accompanied by a marked reduction in HDL-C levels (Figure 2F). These alterations confirmed the successful establishment of the hyperlipidemia model. RES treatment effectively ameliorated these lipid abnormalities, particularly in the RES-M and RES-H groups.

### 3.2. RES Attenuated Hepatic Lipid Accumulation and Inflammation in Hyperlipidemia Mice

**Figure 3:**
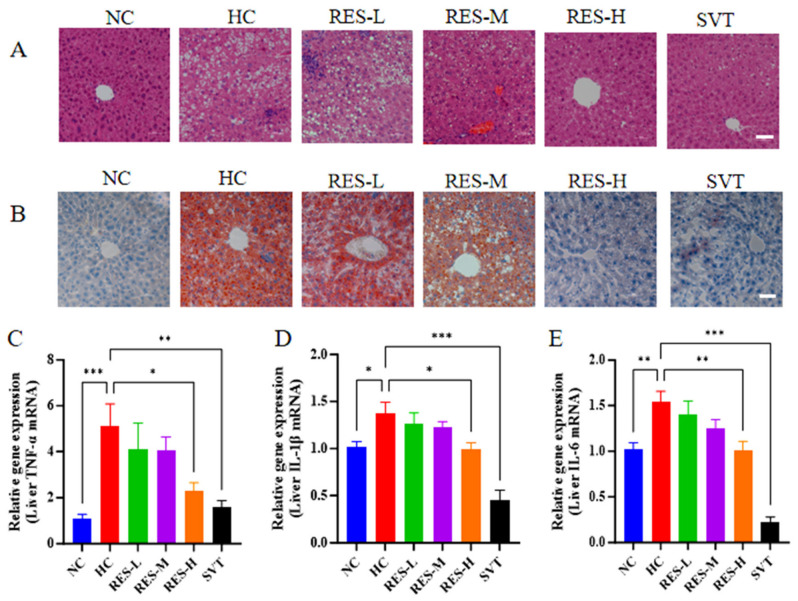
Resveratrol treatment ameliorated hepatic injury, lipid accumulation, and inflammation in hyperlipidemic mice. (**A**) Hematoxylin and eosin staining of liver tissues (scale bar: 50 µm). (**B**) Oil Red O staining of liver tissues (scale bar: 50 µm). (**C**) Hepatic tumor necrosis factor-α mRNA expression. (**D**) Hepatic interleukin-1β (IL-1β) mRNA expression. (**E**) Hepatic IL-6 mRNA expression. Data are presented as mean ± standard error of the mean (*n* = 6). * *p* < 0.05, ** *p* < 0.01, and *** *p* < 0.001.

To investigate whether RES administration could reduce hepatic lipid accumulation, histopathological examination of liver tissues was performed (Figure 3). H&E staining revealed that hepatocytes in the NC group exhibited normal morphology and orderly arrangement. In contrast, liver tissues from the HC group displayed disorganized hepatic architecture with numerous lipid vacuoles of varying sizes, indicative of hepatic steatosis and focal inflammatory infiltration. Following RES treatment, the number and size of hepatic lipid vacuoles were markedly reduced, and cellular boundaries became more clearly defined compared with those in the HC group (Figure 3A). Similarly, ORO staining demonstrated almost no lipid droplet accumulation in the NC group, whereas extensive red-stained lipid droplets were observed in the HC group, indicating substantial hepatic fat deposition. Compared with the HC group, both RES-treated groups and the positive control group exhibited significantly reduced lipid accumulation (Figure 3B). These findings demonstrated that RES effectively alleviated hepatic steatosis and inflammatory injury induced by HFD feeding. Consistent with previous studies, RES exhibited protective effects against HFD-induced hepatic steatosis and hepatocellular ballooning degeneration [15]. Furthermore, hepatic inflammatory responses were evaluated by measuring the expression of pro-inflammatory cytokines. Compared with the NC and RES-treated groups, mice in the HC group showed significantly elevated hepatic expression levels of tumor necrosis factor-α (TNF-α), interleukin-1β (IL-1β), and IL-6. In contrast, treatment with high-dose RES and simvastatin significantly reduced the expression of IL-1β and IL-6 (Figure 3C–E). These results suggested that RES protected the liver from hyperlipidemia-induced injury by improving the pro-inflammatory hepatic microenvironment.

### 3.3. Attenuated Ileal Injury and Inflammation in Hyperlipidemia Mice

**Figure 4:**
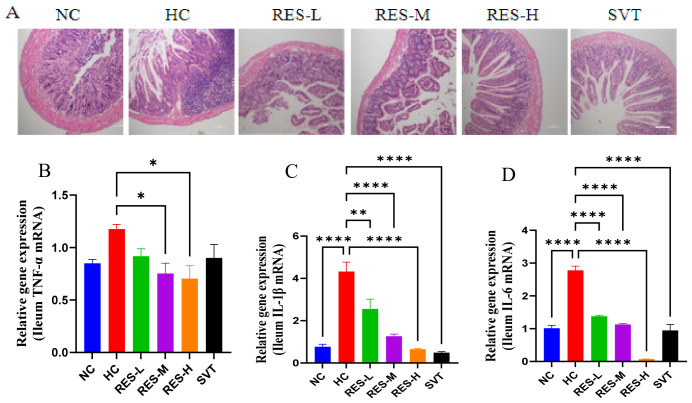
Resveratrol treatment improved ileal injury and inflammation in hyperlipidemic mice. (**A**) hematoxylin and eosin staining of ileal tissues (scale bar: 100 µm). (**B**) Ileal tumor necrosis factor-α mRNA expression. (**C**) Ileal interleukin-1β (IL-1β) mRNA expression. (**D**) Ileal IL-6 mRNA expression. Data are presented as mean ± standard error of the mean (*n* = 6). * *p* < 0.05, ** *p* < 0.01, and **** *p* < 0.0001.

Long-term consumption of an HFD not only disrupts intestinal barrier integrity but also induces intestinal inflammation [16]. H&E staining revealed substantial histopathological alterations in the ileum of HFD-fed mice, including irregular villus morphology, crypt disorganization, and thinning of the basal layer (Figure 4A). In contrast, treatment with RES or simvastatin markedly improved intestinal morphology, as evidenced by restoration of villus architecture, improved crypt organization, and increased basal layer thickness. To further assess intestinal inflammation, the mRNA expression levels of inflammatory cytokines in ileal tissues were quantified (Figure 4B–D). The results demonstrated significantly increased expression levels of IL-1β and IL-6 in the HC group, indicating the presence of pronounced intestinal inflammation. RES treatment significantly reduced the expression of these inflammatory mediators, thereby alleviating ileal inflammation.

### 3.4. RES Alleviated BA Dysregulation in Hyperlipidemia Mice

**Figure 5:**
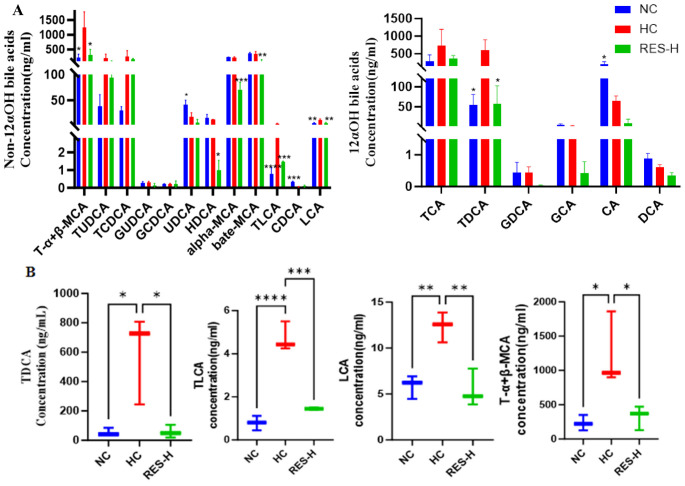
Resveratrol treatment altered bile acid (BA) metabolism in hyperlipidemic mice. (**A**) Serum concentrations of major BAs. (**B**) Differentially expressed serum BAs among groups. Data are presented as mean ± standard error of the mean (*n* = 3). * *p* < 0.05, ** *p* < 0.01, *** *p* < 0.001 and **** *p* < 0.0001.

BAs are critical regulators of metabolic homeostasis and are involved not only in lipid digestion and absorption but also in the pathogenesis of metabolic disorders. To investigate the effects of RES on BA metabolism, a UPLC-MS/MS-based targeted metabolomics approach was employed to qualitatively and quantitatively analyze serum BA profiles (Figure 5). A total of 18 BAs were identified, including the 12α-hydroxylated BAs (12α-OH BAs), namely TCA, taurodeoxycholic acid (TDCA), glycodeoxycholic acid, glycocholic acid, cholic acid (CA), and deoxycholic acid, as well as the non-12α-OH BAs, including tauro-(α + β)-muricholic acid [T-(α + β)-MCA], tauroursodeoxycholic acid, glycoursodeoxycholic acid, glycochenodeoxycholic acid, ursodeoxycholic acid, hyodeoxycholic acid, α-muricholic acid, β-muricholic acid, taurolithocholic acid (TLCA), chenodeoxycholic acid, and lithocholic acid (LCA). BAs are synthesized in the liver through either the classical pathway or the alternative pathway. The classical pathway predominantly generates 12α-OH BAs, whereas the alternative pathway primarily produces non-12α-OH BAs. Increasing evidence suggests that the alternative pathway plays an important role in regulating lipid metabolism, cholesterol homeostasis, glucose metabolism, and energy balance [17]. In the present study, no significant differences were observed in most serum 12α-OH BA levels among the experimental groups. In contrast, RES treatment significantly reduced the levels of several non-12α-OH BAs compared with those in the HC group (Figure 5A). Four BAs exhibiting the most pronounced differences were identified, including TDCA, TLCA, LCA, and T-(α + β)-MCA (Figure 5B). Compared with the NC group, serum concentrations of these BAs were significantly increased in the HC group. Notably, treatment with high-dose RES markedly reduced the levels of these altered BAs. Collectively, these findings demonstrated that HFD feeding disrupted bile acid homeostasis, whereas RES effectively ameliorated these metabolic disturbances. The prominent effects of RES on non-12α-OH BAs suggest that its lipid-lowering effects may be mediated, at least in part, through modulation of bile acid synthesis via the alternative pathway.

### 3.5. RES Alleviated Gut Microbiota Dysbiosis in Hyperlipidemia Mice

**Figure 6:**
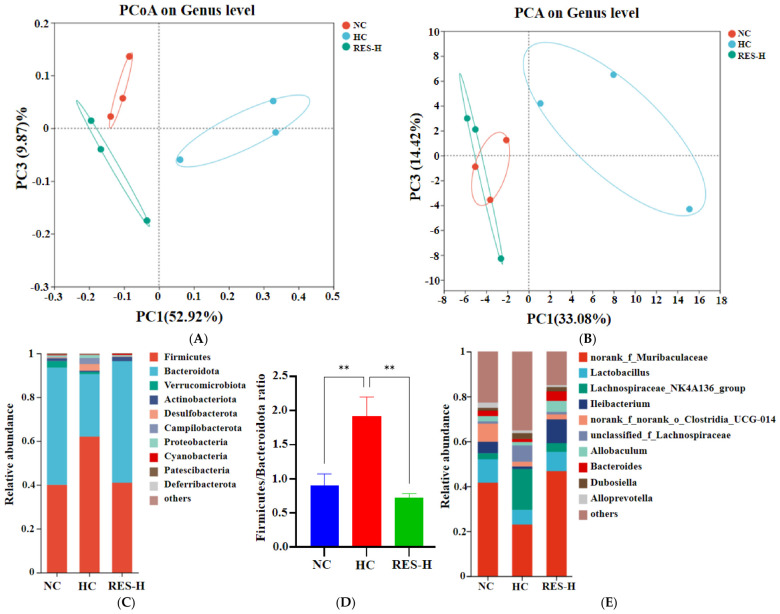

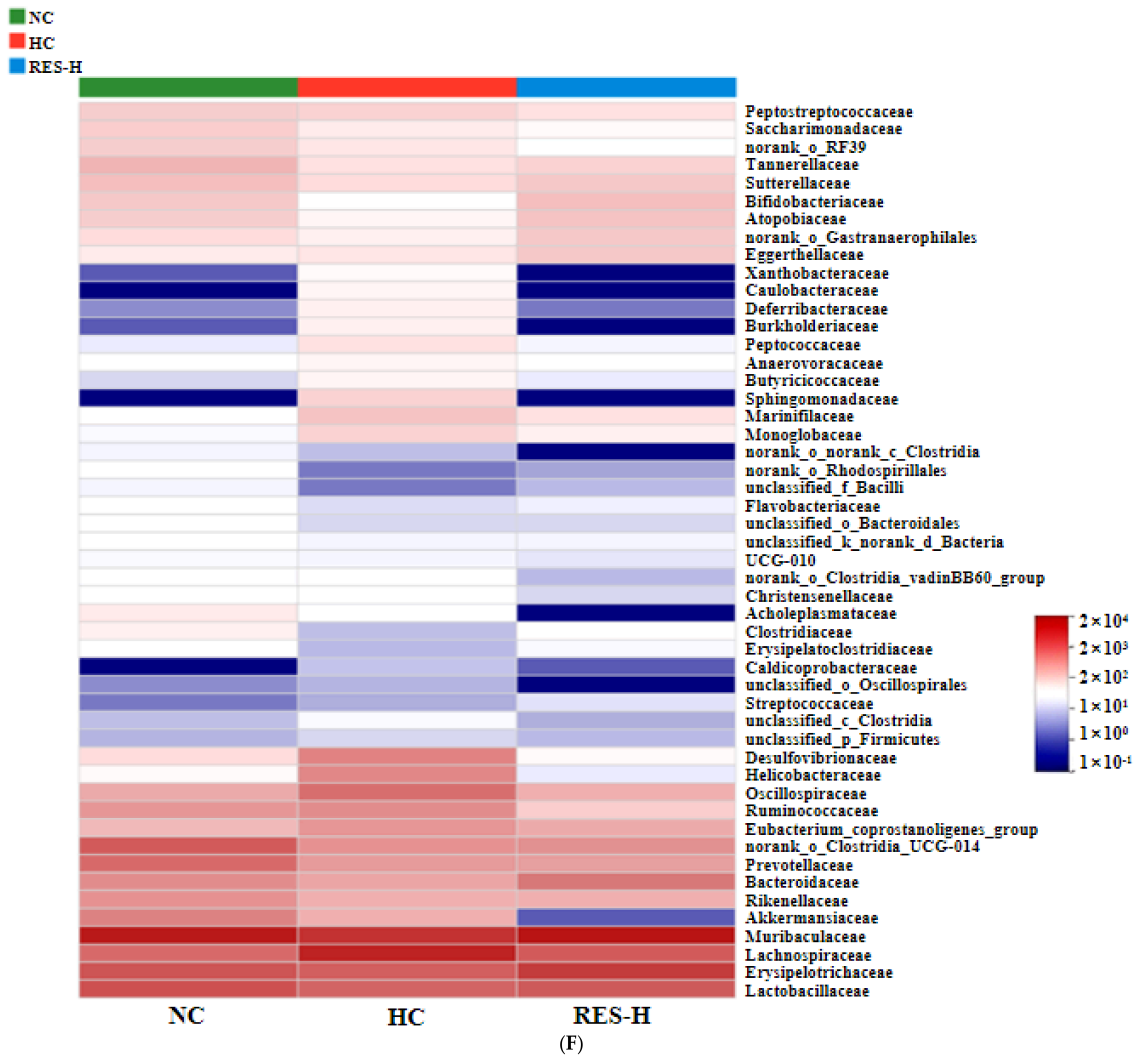
Effects of resveratrol on gut microbial composition in hyperlipidemic mice. (**A**) PCoA plot based on Bray–Curtis distances. (**B**) Principal component analysis plot based on operational taxonomic units. (**C**) Relative abundance of gut microbiota at the phylum level. (**D**) Firmicutes/Bacteroidota ratio. (**E**) Relative abundance of gut microbiota at the genus level. (**F**) Heatmap of family-level microbial composition. Data are presented as mean ± standard error of the mean (*n* = 3). ** *p* < 0.01.

Because BA metabolism is closely associated with the gut microbiota [6], 16S rRNA gene sequencing was performed to evaluate alterations in intestinal microbial composition and abundance. UniFrac distance-based principal coordinate analysis (PCoA) revealed clear separation of microbial communities among the experimental groups, indicating that both HFD feeding and RES treatment significantly altered gut microbial structure (Figure 6A). Similarly, principal component analysis (PCA) demonstrated that the microbial community composition of the RES-H group clustered more closely with that of the NC group than with that of the HC group (Figure 6B). At the phylum level, the predominant bacterial taxa included *Firmicutes, Bacteroidota*, *Verrucomicrobiota*, *Actinobacteriota*, *Desulfobacterota*, *Campylobacterota*, and *Proteobacteria* (Figure 6C). Compared with the NC group, the HC group exhibited a significantly increased *Firmicutes/Bacteroidetes* (F/B) ratio (*p* < 0.01), whereas high-dose RES treatment effectively reversed this alteration (Figure 6D). At the genus level, the dominant bacterial taxa included *non_f_Muribaculaceae, Lactobacillus*, *Lachnospiraceae_NK4A136_group*, *Ileibacterium*, *norank_f_norank_o_Clostridia_UCG-014*, *unclassified_f_Lachnospiraceae*, *Allobaculum*, and *Bacteroides*. Compared with the NC group, the HC group exhibited reduced abundances of *non_f_Muribaculaceae*, *Lactobacillus*, *Ileibacterium*, and *Bacteroides*, while the abundances of *Lachnospiraceae_NK4A136_group* and *unclassified_f_Lachnospiraceae* were significantly increased. Conversely, RES treatment increased the abundances of beneficial bacterial genera and reduced the abundance of potentially harmful taxa (Figure 6E). Heatmap analysis at the family level demonstrated that the microbial composition of the RES-H group closely resembled that of the NC group. The most notable differences were observed in the families *Sphingomonadaceae*, *Xanthobacteraceae*, *Burkholderiaceae*, and *Caulobacteraceae*, all of which belong to the phylum *Proteobacteria*. Specifically, the HC group exhibited markedly higher abundances of *Proteobacteria*-related taxa than either the NC or RES-H groups (Figure 6F). These findings collectively demonstrated that RES effectively modulated gut microbial composition and partially restored HFD-induced gut microbiota dysbiosis.

**Figure 7:**
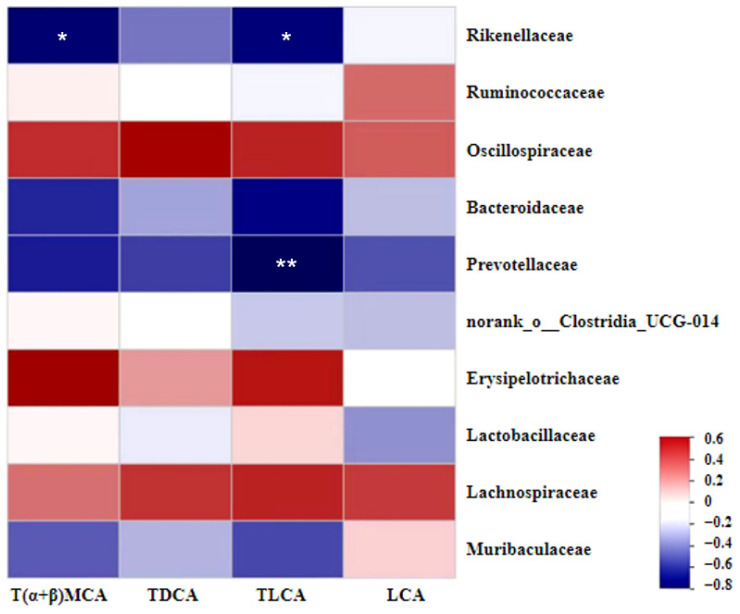
Heatmap illustrating Spearman’s correlations between significantly altered serum bile acids and gut microbial taxa. * *p* < 0.05, ** *p* < 0.01.

To further explore the interaction between gut microbiota and bile acid metabolism, Spearman’s correlation analysis was performed (Figure 7). The results demonstrated that differential BAs were positively correlated with potentially harmful bacterial taxa, including Lachnospiraceae and Oscillospiraceae. In contrast, Prevotellaceae and Rikenellaceae were negatively correlated with several differential BAs, particularly T-(α+β)-MCA and TLCA. Both Prevotellaceae and Rikenellaceae belong to the phylum Bacteroidota and have been reported to regulate adiposity through the production of short-chain fatty acids (SCFAs), particularly acetate and propionate [18,19].

## 4. Discussion

The present study systematically investigated the protective effects of RES against HFD-induced metabolic disturbances in mice, encompassing body weight regulation, lipid metabolism, hepatic injury, BA homeostasis, and gut microbiota composition. Collectively, our findings demonstrate that RES exerts multifaceted protective effects against HFD-induced hyperlipidemia and its associated metabolic complications. Specifically, RES significantly attenuated HFD-induced body weight gain and improved dyslipidemia, as evidenced by reductions in serum TC and LDL-C levels, together with an increase in HDL-C concentrations. Hepatic steatosis and inflammation are hallmark features of HFD-induced metabolic syndrome, and RES treatment markedly alleviated these pathological alterations. Furthermore, RES effectively ameliorated intestinal inflammation, suggesting that its beneficial effects extend beyond systemic lipid metabolism to include protection of intestinal homeostasis and barrier integrity.

BAs are central regulators of lipid metabolism, and disturbances in BA homeostasis are closely associated with the development and progression of metabolic disorders. In the present study, serum BA profiles were characterized using UPLC-MS/MS-based targeted metabolomics. The results demonstrated that RES predominantly modulated non-12α-OH BAs rather than 12α-OH BAs in HFD-fed mice. Specifically, RES significantly reduced the serum concentrations of TDCA, TLCA, LCA, and T-(α+β)-MCA, all of which were elevated following HFD. TDCA, a secondary BA, has been reported to exhibit carcinogenic potential and has been implicated in intestinal barrier dysfunction and mucosal injury [20]. Therefore, the elevated TDCA levels observed in HFD-fed mice may have contributed to both hyperlipidemia and intestinal barrier impairment. TLCA, a potent agonist of the Takeda G protein-coupled receptor 5, has been shown to regulate lipid metabolism through gut–brain signaling pathways and may influence adipose tissue thermogenesis and energy expenditure [21,22]. LCA, one of the most abundant secondary BAs, is considered a highly cytotoxic endogenous metabolite generated by gut microbial metabolism and has been implicated in hepatocellular injury and liver dysfunction [23]. Accordingly, the hepatic injury observed in the HFD group may be partially attributable to elevated circulating LCA concentrations. T-(α + β)-MCA is a murine-specific conjugated BA that functions as an endogenous antagonist of the farnesoid X receptor (FXR) and can be rapidly hydrolyzed by microbial bile salt hydrolases in the intestine [24]. In the present study, RES administration significantly reduced serum T-(α + β)-MCA levels, which may relieve FXR inhibition and promote FXR activation. Activation of intestinal FXR stimulates fibroblast growth factor 15 secretion, which subsequently suppresses hepatic BA synthesis through negative feedback inhibition of key BA synthetic enzymes, ultimately reducing BA production [25].

The gut microbiota plays an essential role in regulating BA synthesis, transformation, and signaling. Conversely, BAs act as important signaling molecules that shape gut microbial composition and function, establishing a bidirectional regulatory relationship between gut microorganisms and host metabolism [26,27]. Consistent with this concept, 16S rRNA gene sequencing revealed substantial differences in gut microbial composition among the experimental groups. Compared with the HFD control group, high-dose RES treatment significantly increased the relative abundances of *non_f_Muribaculaceae*, *Lactobacillus*, *Ileibacterium*, and *Bacteroides*, while reducing the abundances of *Lachnospiraceae_NK4A136_group* and unclassified*_f_Lachnospiraceae*. Members of the family Muribaculaceae are generally considered beneficial intestinal microorganisms that contribute to carbohydrate metabolism, promote lipolysis, and improve insulin sensitivity [28]. *Lactobacillus* species are well known for their ability to strengthen intestinal barrier integrity, enhance nutrient utilization, promote BA and SCFA metabolism, and protect intestinal tissues from environmental damage [29]. Similarly, *Ileibacterium* has been identified as a beneficial bacterial genus associated with improved carbohydrate metabolism and restoration of gut microbial homeostasis under HFD conditions [30]. Moreover, RES treatment increased the abundance of *Bacteroides*, a genus involved in mucin utilization and intestinal immune regulation, which may contribute to attenuation of intestinal inflammation by reducing harmful host–microbe interactions and improving mucosal barrier function [31]. In contrast, the abundance of *Lachnospiraceae_NK4A136_group* has been reported to increase in several pathological conditions, including inflammatory bowel disease, hepatic steatosis, and metabolic dysfunction [32,33].

The reduction of this taxon following RES administration therefore further supports the beneficial effects of RES on metabolic homeostasis. To further elucidate the interactions between gut microbiota and BA metabolism, Spearman correlation analysis was performed to assess associations between differential bacterial taxa and BA profiles. The results demonstrated that the relative abundances of differential BAs were positively correlated with potentially detrimental bacterial taxa, including members of the families Lachnospiraceae and Oscillospiraceae. Importantly, elevated concentrations of TDCA, TLCA, and LCA have been shown to impair intestinal barrier integrity, induce metabolic inflammation, and disrupt both microbial and host metabolic functions [34]. The enrichment of Lachnospiraceae and Oscillospiraceae may further aggravate BA dysregulation by altering microbial BA biotransformation processes and reducing BA metabolic efficiency [35,36]. In contrast, enrichment of beneficial taxa such as Prevotellaceae and Rikenellaceae may contribute to metabolic homeostasis through the production of SCFAs, regulation of energy harvest, and modulation of BA synthesis and excretion, thereby alleviating hypercholesterolemia [37,38]. Based on these correlative observations, shifts within the gut microbiota-BA axis might correlate with resveratrol’s hypolipidemic activity. Targeted interventions on specific gut microbial populations and bile acid metabolic profiles may warrant further investigation as a candidate approach to relieve hyperlipidemia and its concomitant metabolic complications.

## 5. Conclusions

This study investigated the effects of RES on HFD-induced hyperlipidemia in mice. Hyperlipidemia is a common metabolic disorder and a major risk factor for cardiovascular disease, and is frequently accompanied by gut microbial dysbiosis, disrupted BA metabolism, and impaired intestinal barrier function. Our findings demonstrated that RES markedly improved serum lipid profiles and alleviated inflammation and hepatic lipid accumulation in hyperlipidemic mice, which is consistent with previous studies. Furthermore, RES attenuated intestinal injury and significantly modulated both BA metabolism and gut microbiota composition. The gut microbiota–BA regulatory network may represent the central mechanism underlying the protective effects of RES against hyperlipidemia. Taken together, these findings provide experimental evidence supporting the therapeutic potential of RES for hyperlipidemia and related metabolic disorders and suggest that modulation of the gut microbiota–BA axis may represent a promising strategy for the treatment of metabolic diseases.

## Figures and Tables

**Table 1:** Primer sequences used for quantitative real-time polymerase chain reaction analysis.

Genes	Forward Primer	Reverse Primer
β-actin	5′-GGCTGTATTCCCCTCCATCG-3′	5′-CCAGTTGGTAACAATGCCATGT-3′
IL-1β	5′-TTCGAGGCACAAGGCACAACA-3′	5′-AGGTCCTGGAAGGAGCACTTCA-3′
TNF-α	5′-ATGTCTCAGCCTCTTCTCATTC-3′	5′-GCTTGTCACTCGAATTTTGAGA-3′
IL-6	5′-AGCGATGATGCACTGTCAGA-3′	5′-GGAACTCCAGAAGACCAGAGC-3′

## Data Availability

The original contributions presented in this study are included in the article. Further inquiries can be directed to the corresponding author.

## Abbreviations

RES, resveratrol; BAs, bile acids; HFD, high-fat diet; TC, total cholesterol; TG, triglycerides; HDL-C, high-density lipoprotein cholesterol; LDL-C, low-density lipoprotein cholesterol; NC, normal control group; CMC-Na, sodium carboxymethyl cellulose; RES-L, low-dose resveratrol group; RES-M, medium-dose RES group; RES-H, high-dose resveratrol group; SVT, simvastatin; HC, high-fat control group; qPCR, quantitative polymerase chain reaction; TNF-α, tumor necrosis factor-α; IL-1β, interleukin-1β; IL-6, interleukin-6; H&E staining, hematoxylin and eosin staining; TCA, taurocholic acid; TDCA, taurodeoxycholic acid; GDCA, glycodeoxycholic acid; GCA, glycocholic acid; CA, cholic acid; DCA, deoxycholic acid; T-(α+β)-MCA, tauro-(α+β)-muricholic acid; TUDCA, tauroursodeoxycholic acid; GUDCA, glycoursodeoxycholic acid; GCDCA, glycochenodeoxycholic acid; UDCA, ursodeoxycholic acid; HDCA, hyodeoxycholic acid; α-MCA, α-muricholic acid; β-MCA, β-muricholic acid; TLCA, taurolithocholic acid; CDCA, chenodeoxycholic acid; LCA, lithocholic acid; 12α-OH BAs, 12α-OH BAs; and non-12α-OH BAs, non-12α-OH BAs.
